# MiRNAs from *DLK1-DIO3* Imprinted Locus at 14q32 are Associated with Multiple Sclerosis: Gender-Specific Expression and Regulation of Receptor Tyrosine Kinases Signaling

**DOI:** 10.3390/cells8020133

**Published:** 2019-02-08

**Authors:** Natalia Baulina, German Osmak, Ivan Kiselev, Ekaterina Popova, Alexey Boyko, Olga Kulakova, Olga Favorova

**Affiliations:** Pirogov Russian National Research Medical University, Moscow 117997, Russia; german.osmak@gmail.com (G.O.); kiselev.ivan.1991@gmail.com (I.K.); ani_retake1@mail.ru (E.P.); boykoan13@gmail.com (A.B.); olga.koulakova@gmail.com (O.K.); olga.favorova@gmail.com (O.F.)

**Keywords:** relapsing-remitting multiple sclerosis, miRNA, *DLK1-DIO3* imprinted locus, gender specificity, network-based enrichment analysis, receptor tyrosine kinase

## Abstract

Relapsing-remitting multiple sclerosis (RRMS) is the most prevalent course of multiple sclerosis. It is an autoimmune inflammatory disease of the central nervous system. To investigate the gender-specific involvement of microRNAs (miRNAs) in RRMS pathogenesis, we compared miRNA profiles in peripheral blood mononuclear cells separately in men and women (eight RRMS patients versus four healthy controls of each gender) using high-throughput sequencing. In contrast to women, six downregulated and 26 upregulated miRNAs (*p_ad_*_j_ < 0.05) were identified in men with RRMS. Genes encoding upregulated miRNAs are co-localized in *DLK1-DIO3* imprinted locus on human chromosome 14q32. Reverse transcription quantitative polymerase chain reaction (RT-qPCR) analysis was performed in independent groups of men (16 RRMS patients and 10 healthy controls) and women (20 RRMS patients and 10 healthy controls). Increased expression of miR-431, miR-127-3p, miR-379, miR-376c, miR-381, miR-410 and miR-656 was again demonstrated in male (*p_ad_*_j_ < 0.05), but not in female RRMS patients. At the same time, the expression levels of these miRNAs were lower in healthy men than in healthy women, whereas in RRMS men they increased and reached or exceeded levels in RRMS women. In general, we demonstrated that expression levels of these miRNAs depend both on “health–disease” status and gender. Network-based enrichment analysis identified that receptor tyrosine kinases-activated pathways were enriched with products of genes targeted by miRNAs from *DLK1-DIO3* locus. These results suggest the male-specific involvement of these miRNAs in RRMS pathogenesis via regulation of PI3K/Akt signaling.

## 1. Introduction

Multiple sclerosis (MS) is an autoimmune inflammatory disease of central nervous system, which is characterized by demyelination and axon damage caused by inflammatory attacks. By studying the most common MS course, the relapsing-remitting MS (RRMS), which is characterized by recurrent acute exacerbations followed by partial or complete recovery [1], one can focus on general mechanisms that underlie the complex pathologic processes in MS.

Long-term studies of genetic susceptibility to RRMS couldn’t explain a substantial proportion of the observed heritability. In today’s knowledge this is related to the minor effects of genes, which are not directly associated with the disease. These genes could be integrated in networks, regulated by miRNAs—small (19–24 nucleotides) non-coding RNAs. MiRNA inhibits target mRNAs’ expression through the complete or incomplete complementary binding of the miRNA seed region (6–8 nucleotide sequence) mainly with the 3′-untranslated region of mRNA [2]. MiRNAs were shown to mediate the normal functioning of the immune system and, moreover, to regulate the autoimmune inflammatory processes, including MS [3,4]. One of the most effective approaches in searching for novel MS-related miRNAs is high-throughput sequencing (next generation sequencing, NGS), which allows to examine the expression of all miRNAs in the analyzed biological material. Thus far, only a few studies applied this approach to identify miRNA signatures in RRMS, focusing mostly on the expression of miRNAs in serum or in whole blood of patients and healthy individuals [5,6,7,8,9].

Similar to other autoimmune diseases, RRMS is more prevalent in women than in men [10]. Recent studies demonstrated gender differences in miRNA expression in MS [11,12], which raised the question of miRNA involvement in different molecular processes promoting MS in men and women. Using high-throughput sequencing, we analyzed the miRNA profiles looking for MS-related miRNAs separately in men and women. We have chosen peripheral blood mononuclear cells (PBMC) as an informative and easily accessible biological material. To estimate the functional role of differentially expressed miRNAs, we applied network-based enrichment analysis.

## 2. Materials and Methods

### 2.1. Patients and Controls

52 unrelated patients with RRMS and 28 healthy controls (HCs) of comparable age and sex were enrolled in the study. Among RRMS patients 24 were in clinical relapse (24–36 h after relapse manifestation and before the first corticosteroid administration) and 28 were in remission (clinically stable for at least 6 months). All RRMS patients fulfilled the McDonald criteria for MS diagnosis (2010 revisions) [13], were treatment-naive and were free from any other inflammatory disorders. A group of HCs was formed from volunteers free of significant acute or chronic inflammatory diseases and not on any medications. Demographic and clinical characteristics of all the subjects included in the study at the stages of sequencing (16 RRMS patients and eight HCs) and subsequent validation (36 RRMS patients and 20 HCs) are shown in Table 1. No significant differences in demographic and clinical characteristics were observed when RRMS patients were stratified by gender (data not shown). Written informed consent was obtained from all patients prior to their inclusion in this study. The study was approved by the Ethics Committee of the Pirogov Russian National Research Medical University (Decision #139 from 10/11/2014). 

### 2.2. RNA Isolation

PBMC were isolated using Ficoll-Hypaque density gradient method (Sigma-Aldrich, St. Louis, MO, USA) within 3 h of sampling. RNA was isolated using miRNeasy Mini Kit (Qiagen, Hilden, Germany). The total RNA concentration was measured with a NanoDrop-2000 spectrophotometer and the RNA integrity was assessed by QIAxcel Advanced System (Qiagen, Hilden, Germany). Samples with a RNA integrity number (RIN) value above eight were included in subsequent experiments.

### 2.3. RNA-seq Data Analysis

Small RNA libraries were constructed using TruSeq® Small RNA Library Prep Kit (Illumina, San Diego, CA, USA) following manufacturer instructions. Samples were individually barcoded and sequenced in multiplexed pools each containing four samples using the 36 bp fragment protocol on Illumina MiSeq platform. The sequencing generated an average of 15M reads. All miRNA-sequencing data are deposited in the international public repository, Gene Expression Omnibus, under accession identification as GSE124900 [14].

### 2.4. Reverse Transcription Quantitative Polymerase Chain Reaction (RT-qPCR)

Mature miRNA TaqMan assays (ThermoFisher Scientific, Waltham, MA, USA) were used to validate differential expression of miRNAs based on RNA-seq data analysis. The small RNA RNU6B was used as endogenous control due to the smallest variance in its expression in all samples. MiRNA differential expression was calculated using the ΔΔCt method [15].

### 2.5. Statistical Analysis

R programming language was used to perform all statistical analyses. The sample size calculation for compared groups was performed using the pwr library based on statistical significance level of 0.05, power of 0.8 and the expected effect size of more than 2. The sequencing data analysis on miRNA expression between RRMS and HCs and between patients in remission and patients in relapse separately in men and women was conducted using the limma package [16]. The False Discovery Rate (FDR) procedure of Benjamini-Hochberg was used to adjust for multiple testing correction (*p_adj_*) [17]. MiRNA considered to be differentially expressed if *p_adj_* was less 0.05 and logarithm of fold change (Log_2_FC) value was less −1 or more 1. The interaction between “health–disease” status and gender that determines the expression levels of selected miRNAs in (−ΔCt) values was estimated using two-way ANOVA. The assumptions in ANOVA were checked based on Shapiro-Wilk and Levene’s tests; to fulfill these assumptions the data were then Z scale-transformed. The significance of interaction beased in ANOVA test was adjusted for multiple testing correction using Benjamini-Hochberg procedure. The post-hoc analysis was performed using Tukey’s HSD (honestly significant difference). Threshold for statistical significance was equal to *p_adj_* < 0.05.

### 2.6. Network-Based Enrichment Analysis 

To estimate the functional role of differentially expressed miRNAs, the over-representation analysis was carried out based on the representation of differentially expressed miRNAs’ target genes in a specific signaling pathway from the Reactome pathway database of the Molecular Signatures Database (MSigDB) [18] using a method described in [19]. Reactome pathways, which were significantly overrepresented by miRNA target genes, were set as nodes; two nodes were connected by edge when overlap between their gene sets did exist. Edge weight was equal to ratio of cardinality of gene set’ overlaps between two pathways to the union of gene sets of these two pathways. A network of overlaps was built and analyzed using library NetworkX version 2.1 for Python 3.7 [20]. The network was visualized using prefuse force directed layout by edge weight in Cytoscape version 3.6.1 [21]. 

## 3. Results

### 3.1. NGS Screening of Differentially Expressed miRNAs and the Following Validation

We performed a small RNA high-throughput sequencing in PBMC of 16 RRMS patients (eight men and eight women) and 8 HCs (four men and four women). Based on statistical estimation, a sample of four people is sufficient to carry out the present study. Тaking into account the specificity of disease course in men and women, we applied gender-based approach. For searching MS-associated miRNAs we compared eight male RRMS patients to four healthy men and identified 114 differentially expressed miRNAs (*p* < 0.05; −1 < Log_2_FC > 1) of which 32 miRNAs passed threshold for multiple corrections (*p*_adj_ < 0.05). Among them, six miRNAs were downregulated (Log_2_FC < −1), while the other 26 miRNAs were upregulated (Log_2_FC > 1) in RRMS patients (Table 2). In case of miRNA profiling in eight women with RRMS and four healthy women, none of the 46 differentially expressed miRNAs passed the FDR threshold (Table S1). Thus, substantial changes in miRNA expression profiles were found out only in men.

**Figure 1:**
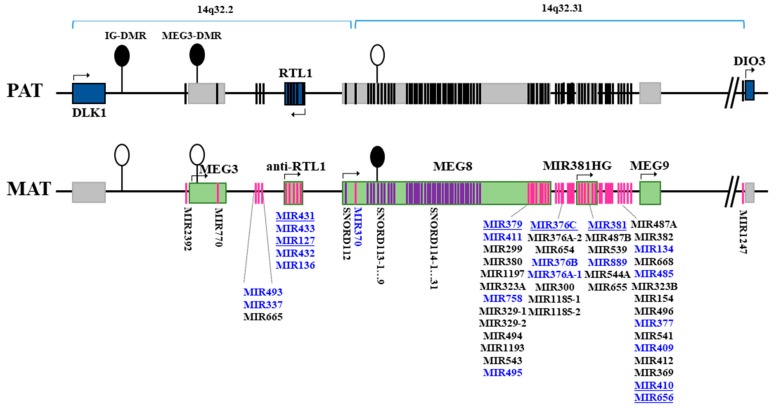
Localization of miRNA genes with increased expression in male patients with relapsing-remitting multiple sclerosis (in blue color) within *DLK1-DIO3* imprinted locus on human chromosome 14q32. The schematic representation of the locus was constructed based on current concept of its structure (according to Human Genome Assembly GRCh38.p12 [22]). Groups of miRNA genes located in close proximity to each other or referred to one structural unit of genome are pooled in lists. Altogether, this locus harbors two large clusters of miRNA genes (10 miRNA genes in 14q32.2 and 44 miRNA genes in 14q32.31). Underlined miRNAs were further taken for validation analysis. The *DLK1-DIO3* imprinted locus contains three paternally-expressed protein-coding genes: *DLK1* (Delta-like homolog 1), which is located at the 5′-end and encodes for a protein involved in the Notch signaling pathway [23], *DIO3* (type III iodothyronine deiodinase) located at the 3′-end and involved in controlling thyroid hormone homeostasis [24] and *RTL1* (Retrotransposon Gag Like 1). Fully complementary antisense transcript anti-*RTL1* is expressed from the maternal chromosome and acts as a repressor for *RTL1*. The locus also contains long non-coding RNAs genes (*MEG3*, *MEG8*, *MIR381HG*, *MEG9*), coming from the maternal chromosome. *MEG8* harbors a tandemly repeated array of the C/D-box snoRNA family, namely *SNORD112*, *SNORD113*, *SNORD114*, consisting of one, nine and 31 paralogous copies, respectively. The expression of genes is regulated via imprinting control regions which harbor differentially methylated regions (DMRs): the intergenic DMR (IG-DMR), MEG3-DMR and MEG8-DMR. The IG- and MEG3-DMRs are methylated on the paternal and unmethylated on the maternal allele, while the MEG8-DMR is oppositely methylated on the maternal allele [25]. PAT and MAT stand for paternal and maternal chromosomes; DMR—differentially methylated region. Filled ellipses represent methylated DMRs, and unfilled represent unmethylated DMRs. Blue rectangles represent protein-coding paternally-expressed genes, and green—maternally-expressed non-coding genes; gray rectangles represent repressed genes. Black strips indicate for paternal repressed miRNA and SNORD genes; pink and violet strips indicate maternally-expressed miRNA and SNORD genes, respectively.

As can be seen from Table 2, genes encoding downregulated miRNAs in men are located in different chromosomes, whereas all genes of upregulated miRNAs in men were found to be structurally associated by their genomic location on the long arm of chromosome 14 (14q32) in *DLK1-DIO3* imprinted locus. The schematic representation of *DLK1-DIO3* imprinted locus and localization of identified miRNA genes with increased expression in male RRMS patients within this locus are depicted on Figure 1.

As RRMS patients in the study differed by disease activity (remission and relapse), we also compared the miRNA expression patterns between RRMS patients in remission and relapse separately for men and women (each group consisted of four patients). In women, we did not find specific for remission or relapse miRNAs, which passed FDR correction (not shown). In men, miR-1 was found to be upregulated in relapse relative to remission (Log_2_FC = 3.82; *p*_adj_ = 0.002) (Table 2), whereas expression of all other miRNAs including those transcribed from *DLK1-DIO3* imprinted locus was not identified as stage-specific. From this point on, the disease activity was not taken into account.

**Figure 2:**
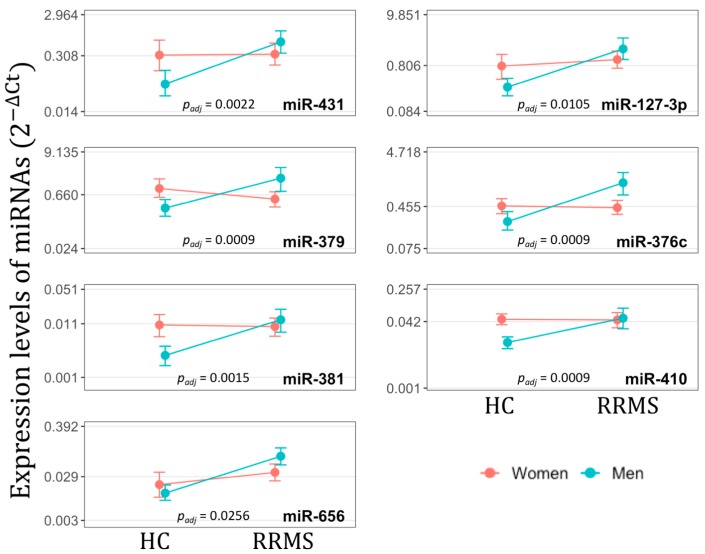
Interaction plots for expression levels (2^−^^ΔCt^) of miR-431, miR-127-3p, miR-379, miR-376c, miR-381, miR-410 and miR-656 according to “health–disease” status and gender of individuals, as determined by RT-*q*PCR. Mean (2^−ΔCt^) values were calculated for healthy controls (HC), i.e., healthy men and healthy women, and for relapsing-remitting multiple sclerosis (RRMS) patients, i.e., RRMS men and RRMS women. The intersection of lines on the plot suggests that there is an interaction effect between “health–disease” status and gender, what is confirmed by the significant *p*_adj_ values.

To confirm high-throughput sequencing data on the gender specificity of differential expression of miRNAs from *DLK1-DIO3* locus in RRMS, we selected miR-431, miR-127-3p, miR-379, miR-376c, miR-381, miR-410 and miR-656 (underlined in Figure 1): miR-431 and miR-127-3p were located in 14q32.2 miRNA cluster, the other five in different structural units of the 14q32.31 miRNA cluster. Reverse transcription quantitative polymerase chain reaction (RT-*q*PCR) analysis was performed in independent groups of men (16 RRMS patients and 10 HCs) and women (20 RRMS patients and 10 HCs). RT-qPCR data were then analyzed by two-way ANOVA to estimate the interplay (interaction) between “health–disease” status and gender that influences miRNA expression. The assumptions of ANOVA were checked using Shapiro-Wilk and Levene’s tests (Table S2). The levels of analyzed miRNAs were lower in healthy men than in healthy women, whereas in RRMS men they increased and reached or exceeded levels in RRMS women (Figure 2). For all miRNAs observed interactions between gender and “health–disease” status were significant, according to ANOVA analysis; after the procedure of Benjamini-Hochberg multiple correction *p*_adj_ ranged from 0.026 to 0.0009. Thus, the impact of “health–disease” status on miRNA expression from *DLK1-DIO3* locus should be qualified in terms of the impact of gender. 

To quantify observed differences between four compared groups we performed post-hoc analysis using Tukey’s HSD (Table S3). Based on this analysis, when compared RRMS to HCs, the expression levels of all miRNAs from *DLK1-DIO3* locus in men were significantly upregulated in RRMS (Log_2_FC ranged from 2.49 to 3.05; *p*_adj_ ranged from 0.00001 to 0.0018) whereas in women these levels did not differ (Table 3), that is in a good accordance with NGS data (Table 2).

**Figure 3:**
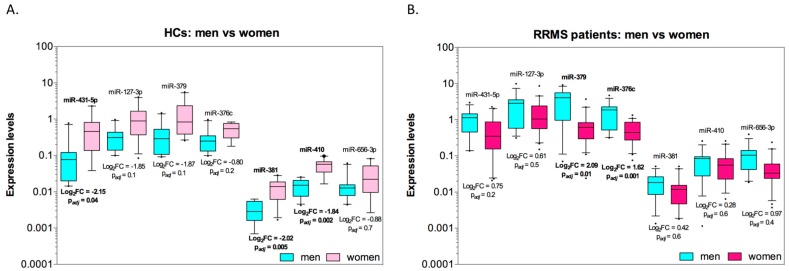
Expression levels of miRNAs in peripheral blood mononuclear cells of men and women (separately in healthy control (HCs) group and relapsing-remitting multiple sclerosis (RRMS) group), as determined by RT-qPCR. Box plots show the minimum, maximum, median and interquartile range for data (Log2 scale) from all individual samples. The points denote outliers. MiRNA expression was calculated relative to endogenous RNU6B using ΔΔCt method. Tukey-test was used to estimate statistical significance between compared groups.

MiRNA expression’ levels for men and women in health and disease, which have been analyzed by post-hoc test, are visualized on Figure 3. The significantly lower expression levels of miR-431-5p, miR-376c and miR-410 were observed in healthy menn than in healthy women: *p*_adj_ was equal to 0.04, 0.0049 and 0.0019, respectively (Figure 3A). In similar comparison in RRMS patients (Figure 3B), levels of all miRNAs expression in men were higher than those in women; for miR-379 and miR-376c, the higher expression was statistically significant (*p*_adj_ was equal to 0.012 and 0.0011, respectively). The absence of significant changes in the expression levels of some miRNAs in combination with unidirectional effects for significant miRNAs may be due to insufficient power of study.

### 3.2. The Analysis of the Functional Role of miRNAs, Differentially Expressed from DLK1-DIO3 Locus in RRMS Pathogenesis

**Figure 4:**
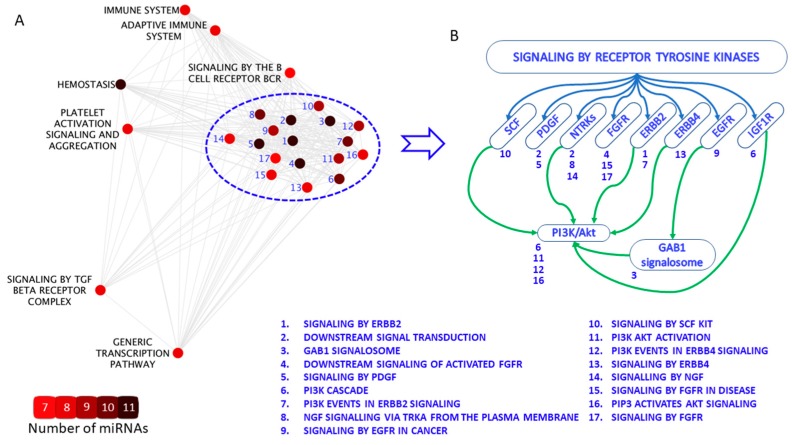
Network-based enrichment analysis of genes targeted by miRNAs differentially expressed from *DLK1-DIO3* locus in male relapsing-remitting multiple sclerosis patients, according to next generation sequencing (NGS) data. (**A**). Network of overlaps for Reactome signaling pathways that significantly enriched with genes targeted by differentially expressed miRNAs (Fisher *p*-value < 0.05). Blue numbering indicates 17 pathways, activated through receptor tyrosine kinases (they are listed in the figure). The color of the nodes represents number of miRNAs, regulating the pathway (ranging from 7 to 11 miRNAs). The more common target genes are shared between two signaling pathways, the closer they placed to each other on the figure. (**B**). The assembly of receptor tyrosine kinase-activated pathways, regulated by identified differentially expressed miRNAs, based on Reactome hierarchy.

The network-based enrichment analysis was carried out to assess the functional role of 26 miRNAs encoded in *DLK1-DIO3* locus, which were upregulated in male RRMS patients according to NGS data (see Table 2). For this, we computed overlaps between the sets of the experimentally confirmed target genes of each of 26 miRNAs (from MiRTaRBase [26]) and the Reactome gene sets (from MSigDB [18]). Reactome sets which were significantly overrepresented by miRNA target genes (*p* < 0.05) were selected for the further analysis. In total, 456 signaling pathways were found to be enriched with target genes of at least one identified miRNA. It emerged that 25% (Q1) of these pathways were enriched with target genes of one miRNA, 50% (Q2)—with target genes of no less than two miRNAs and 75% (Q3), with target genes of no less than three miRNAs. For the following analysis, we chose those pathways, which were enriched with target genes of extreme number of miRNAs, exceeding Q3 + 1.5 interquartile range; as a result, we identified 24 signaling pathways, which were enriched with target genes of more than 6 miRNAs. For these pathways, a network of overlaps was built where signaling pathways (nodes) were connected by edge if overlaps between their gene sets did exist (Figure 4A). Importantly, the constructed network turned out to be a nearly complete graph: 248 edges from 276 edges for complete graph. Of 24 identified pathways 17 were highly overlapped and, as it turned out, belonged among pathways activated through receptor tyrosine kinases (*p* < 0.0001; the calculation was described in Equation S1) [27]. Most of these pathways are regulated by the highest number of miRNAs: from 7 to 11 that is reflected by sizes of nodes on Figure 4A. Based on Reactome hierarchy all of these 17 receptor tyrosine kinase-activated pathways are involved in signal transduction assisted by stem cell factor (SCF), platelet-derived growth factor (PDGF), neurotrophic receptor tyrosine kinase 1 (NTRK1), fibroblast growth factor receptor (FGFR), receptor tyrosine-protein kinase ERBB-2 and ERBB-4, epidermal growth factor receptor (EGFR) and insulin like growth factor 1 receptor (IGF1R) (Figure 4B). The majority of them directly or indirectly (via EGFR-induced GRB2-associated-binding protein 1 activation) regulates PI3K/Akt signaling (green arrows on Figure 4B). Thus, network-based enrichment analysis allowed to single out 17 receptor tyrosine kinase-activated pathways as the most abundantly regulated by miRNAs encoded in *DLK1-DIO3* locus.

## 4. Discussion

Here, we identified novel MS-related miRNAs in PBMC using the hypothesis-free NGS approach. To exclude bias in miRNA expression levels caused by disease modifying therapies, miRNA profiling was performed in treatment-naive RRMS patients. Taking into account hypothetic gender specificity in disease pathogenesis we compared miRNA expression in RRMS patients and HCs separately for men and women. This approach proved to be very informative. MiRNA profiling in men identified 32 MS-related miRNAs, which passed threshold for multiple corrections (*p_adj_* ranged from 0.0017 to 0.048). Results of NGS screening for seven upregulated miRNAs, namely miR-431, miR-127-3p, miR-379, miR-376c, miR-381, miR-410 and miR-656, were validated in an independent sample using RT-qPCR; all selected miRNAs were significantly upregulated in male RRMS patients. At the same time, we did not find well-defined RRMS-specific miRNA expression signatures in women neither using NGS, nor with RT-qPCR (Table 3). Obviously, both increasing the sample number and decreasing the threshold for effect size could lead to detection of additional MS-specific miRNAs in men and/or to identification of any differentially expressed miRNAs in women.

Based on NGS data, we also performed the comparison between RRMS patients in remission and relapse and revealed differences in miRNA expression in men, not in women. Here, only miR-1, which was not previously described as associated with RRMS, was found to be stage-specific in men; it was not among the MS-related miRNAs identified in our study as differentially expressed in men.

Genes of all miRNAs upregulated in male RRMS patients are co-localized in *DLK1-DIO3* locus on chromosome 14. The organization of the *DLK1-DIO3* locus is highly conserved in all mammals and shares similar imprinting regulation of coding and noncoding genes [28]. The *DLK1-DIO3* locus includes the largest in the human genome cluster of 54 miRNAs genes (Figure 1). There are contrary data about the expression mechanisms of miRNA genes from this locus. Some arguments support the hypothesis that all miRNAs are derived from a single polycistronic RNA, sharing a common mechanism of regulation [29]. However, separate enhancers, regulating miRNA expression from 14q32.2 and 14q32.31 clusters in *DLK1-DIO3* locus, were also identified [30,31]. Differences in activity of distinct miRNAs inside one 14q32.2 cluster were also described by other authors [32,33,34], particularly on postnatal, developmental stages, and in adult brains. Moreover, expression levels of not all miRNAs from 14q32 locus were increased in lung adenocarcinoma [35]. Similarly, in our study we observed increased expression of not 54 but 26 miRNAs from both clusters (Log_2_FC > 1 and *p*_adj_ < 0.05). It should be mentioned that NGS analysis identified 25 other miRNAs from *DLK1-DIO3* imprinted locus, from which more than half (15) miRNAs were upregulated in RRMS men with nominal significance (Log_2_FC > 1; *p* < 0.05) (Table S4). Possibly, future investigations will extend the number of MS-associated miRNAs from this locus. In general, all available relevant data highlight that exact ways of miRNA expression from *DLK1-DIO3* locus and mechanisms of its regulation still remain to be uncovered.

The *DLK1-DIO3* locus was shown to be involved in the regulation of different stages of developmental processes. In humans, imprinting abnormalities at the *DLK1-DIO3* locus have been associated with developmental disorders, such as Temple syndrome, caused by maternal uniparental disomy (MatUPD14) (i.e. patients inherit two maternal copies of chromosome 14q32), and Kagami–Ogata syndrome (PatUPD14), caused by paternal uniparental disomy (reviewed in [36]). With that, miRNAs from this imprinting locus were found to be associated with the development of several diseases (reviewed in [37]), such as different types of cancer (reviewed in [36]), cardiovascular diseases [38], autoimmune diseases [39,40] and schizophrenia [41].

Male-specific massive upregulation of miRNAs encoded in imprinted *DLK1-DIO3* locus in MS patients was observed in our study for the first time. However, separate miRNAs from this locus were found to be deregulated during MS earlier. The significant differences in serum levels of miR-127-3p, miR-370-3p, miR-409-3p, miR-432-5p, as well as miR-376c-3p were identified in progressive MS patients’ compared to HCs [5,42]. The expression of miR-433-3p, miR-485-3p and miR-432-5p distinguished between relapsing-remitting and progressive MS patients [5]. Mir-494 was found to be downregulated in T-cells of MS patients when compared to controls [43]. Recently, we demonstrated the upregulation of miR-379-5p in PBMC of remitting MS patients compared to relapsing MS patients [11]. In a good accordance with the results of present study, increased expression of miR-127 and miR-136 after experimental autoimmune encephalomyelitis (EAE) induction in EAE-susceptible rat strains compared to EAE-resistant rat strains was reported; increased expression of other miRNAs from *DLK1-DIO3* locus such as miR-434, miR-541 and miR-369 was also identified in EAE [44].

It is interesting to note that a decrease in expression level of miR-411* from *DLK1-DIO3* imprinted locus was detected in peripheral blood of RRMS patients treated with natalizumab, compared to a treatment-naive RRMS patients [45]. In the context of our results on the increased expression of this miRNA in PBMC of RRMS patients, these data can be considered as the starting point for the study of miRNAs from this locus in terms of prospects of using in clinical implication for RRMS treatment.

In contrast to genes of upregulated miRNAs, genes of identified in our study downregulated miRNAs are distributed over the genome. The data on downregulation of miR-181a and miR-181b are consistent with previous studies, describing miR-181a and -181b suppression in the central nervous system in MS and EAE, as well as in activated lymphocytes [46,47]. Downregulation of miR-3647-3p, miR-3607-3p and miR-330-5p in male RRMS patients was first identified in our study.

Our results showed the validity and effectiveness of the gender-based approach in miRNA expression’ studying. We first established that baseline expression levels of miRNAs from this locus are peculiar to MS patients; these levels preexist in healthy women, whereas in men they are reached or even exceeded only with disease. The origin of this phenomenon is unclear and requires further studying. However, it is tempting to assume that lower incidence of MS in men than in women may be associated with lower expression of miRNAs from *DLK1-DIO3* locus and, at the same time, a more severe disease course in men may be associated with increased expression of these miRNAs in male MS patients.

Recent studies demonstrated gender-specific differences in miRNA expression in various female-predominant autoimmune diseases, including MS [11,12]. Despite the fact that a number of studies were addressed to reviewing the data on sexual dimorphism of miRNA expression [48,49,50], and highlight the important contribution of the X-chromosome and sex hormones in this phenomenon, the nature of the sex-biased miRNA expression is poorly studied. Little is known about miRNAs expression from *DLK1-DIO3* locus in this context. Indeed, the transcription of miR-433 and miR-127 genes encoded within *DLK1-DIO3* locus was found to be regulated by nuclear hormone receptor ERRγ [31]. It was also found that *cis*-miR-eQTL SNP rs4905998 on 14 chromosome influenced allele-specific expression of 16 miRNAs from *DLK1-DIO3* imprinting locus; its proxy SNP rs6575793 was associated with the age at menarche according to genome-wide association study data [51]. Another study demonstrated that imprinted genes more often tend to harbor sex-specific CpGs than non-imprinted; meta-analysis identified significant associations of sex-specific methylation among its CpG regions in *MEG3* gene [52].

Network-enrichment analysis performed on the target genes of differentially expressed miRNAs encoded in the *DLK1-DIO3* locus allowed us to reduce the redundancy among enriched gene sets. From 24 pathways, included in the constructed network, 17 pathways were highly overlapping based on their gene sets and belonged among pathways, activated through receptor tyrosine kinases. These gene sets were reduced to eight “parent” pathways and collocated high up in the Reactome hierarchy [53]. Interestingly, the majority of them directly or indirectly regulate PI3K/Akt signaling; on that basis, miRNAs action is mostly referred to the cell activation, proliferation, metabolism and apoptosis. Recent studies demonstrated the enrichment of the target genes of the miRNAs within *DLK1-DIO3* locus in immune functions [37,54,55]. This is further consistent with the increasing data, showing that alterations in this pathway may result in enhanced susceptibility to autoimmunity [56,57,58].

Although the results described in our study were obtained by two different methods and using two independent samples, further investigation of involvement of miRNAs from *DLK1-DIO3* imprinted locus in MS pathogenesis are imperatively needed, preferably in larger cohorts. Moreover, miRNA expression profiles in cell subtypes other than PBMC should be studied in order to obtain more precise results. Possible directions of research include experimental validation of PI3K/Akt involvement in MS pathogenesis. Altogether, these results could be of use to clarify the role of *DLK1-DIO3* imprinted locus and nature of gender-specific expression of its miRNA genes.

## 5. Conclusions

The involvement of miRNAs encoded in *DLK1-DIO3* imprinted locus in MS pathogenesis was observed for the first time. The upregulation in men was reliably established for miR-431, miR-127-3p, miR-379, miR-376c, miR-381, miR-410 and miR-656 and deemed rather likely for other 19 miRNAs encoded in this locus. At the same time, the expression of these miRNAs in women did not change during MS. Network-based enrichment analysis demonstrated that receptor tyrosine kinase-activated pathways were enriched with products of genes targeted by miRNAs from *DLK1-DIO3* locus, and the majority of these proteins directly or indirectly regulate PI3K/Akt signaling. 

## Figures and Tables

**Table 1:** Demographic and clinical characteristics of the relapsing-remitting multiple sclerosis (RRMS) patients and healthy controls (HCs) included in the study.

	Stable RRMS (Remission)	Acute RRMS (Relapse)	HCs
**Sequencing**			
N	8	8	8
Gender, (M/F)	4/4	4/4	4/4
Age, mean ± SD, (years)	38.9 ± 4.3	35.4 ± 6.8	37.1 ± 6.4
Disease duration, mean ± SD, (years)	5.6 ± 3.2	5.4 ± 3.1	-
EDSS, Expanded Disability Status Scale	2.4 ± 1.0	3.1 ± 1.0	-
**Validation**			
N	20	16	20
Gender, (M/F)	10/10	6/10	10/10
Age, mean ± SD, (years)	34.8 ± 5.0	35.8 ± 4.5	38.1 ± 4.6
Disease duration, mean ± SD, (years)	4.5 ± 2.3	5.3 ± 2.9	-
EDSS, Expanded Disability Status Scale	2.0 ± 0.5	2.7 ± 0.6	-

**Table 2:** MiRNAs, differentially expressed in male relapsing-remitting multiple sclerosis (RRMS) patients when compared to healthy controls (HCs) (A) and in RRMS patients in relapse when compared to RRMS patients in remission (B), identified using high-throughput sequencing.

Number	miRNA	Gene	Localization		Log_2_FC	*p*-value	*p*_adj_
**A. RRMS patients versus HCs**							
Downregulated miRNAs							
1	hsa-miR-3647-3p	*SNORD111B*		16q22.1	−4.15	1.04E-05	0.0017
2	hsa-miR-181a*	*MIR181A2*		9q33.3	−1.50	5.06E-06	0.0017
3	hsa-miR-181a	*MIR181A2*		9q33.3	−1.27	1.73E-05	0.0017
4	hsa-miR-181b	*MIR181B1*		1q32.1	−1.32	8.69E-06	0.0017
5	hsa-miR-3607-3p	*SNORD138*		5q14.3	−3.41	0.00040	0.017
6	hsa-miR-330-5p	*MIR330*		19q13.32	−1.15	0.00075	0.021
Upregulated miRNAs							
1	hsa-miR-431	*MIR431*	14q32.2		1.86	1.21E-05	0.0017
2	hsa-miR-432	*MIR432*	14q32.2		1.71	1.73E-05	0.0017
3	hsa-miR-376c	*MIR376C*	14q32.31		1.86	2.53E-05	0.0021
4	hsa-miR-656	*MIR656*	14q32.31		2.37	3.47E-05	0.0025
5	hsa-miR-409-5p	*MIR409*	14q32.31		5.99	9.94E-05	0.0064
6	hsa-miR-411	*MIR411*	14q32.31		1.54	0.00011	0.0065
7	hsa-miR-376a	*MIR376A-1*	14q32.31		1.99	0.00015	0.0077
8	hsa-miR-377	*MIR377*	14q32.31		2.62	0.00016	0.0077
9	hsa-miR-127-3p	*MIR127*	14q32.2		1.50	0.00023	0.010
10	hsa-miR-410	*MIR410*	14q32.31		1.29	0.00047	0.018
11	hsa-miR-379	*MIR379*	14q32.31		1.69	0.00059	0.020
12	hsa-miR-758	*MIR758*	14q32.31		1.51	0.00065	0.020
13	hsa-miR-889	*MIR889*	14q32.31		1.47	0.00064	0.020
14	hsa-miR-337-3p	*MIR337*	14q32.2		2.25	0.00066	0.020
15	hsa-miR-485-5p	*MIR485*	14q32.31		1.88	0.00075	0.021
16	hsa-miR-485-3p	*MIR485*	14q32.31		1.47	0.00093	0.024
17	hsa-miR-136*	*MIR136*	14q32.2		1.39	0.00093	0.024
18	hsa-miR-433	*MIR433*	14q32.2		1.65	0.0012	0.030
19	hsa-miR-493	*MIR493*	14q32.2		1.31	0.0015	0.032
20	hsa-miR-495	*MIR495*	14q32.31		1.50	0.0015	0.032
21	hsa-miR-337-5p	*MIR337*	14q32.2		2.02	0.0018	0.038
22	hsa-miR-409-3p	*MIR409*	14q32.31		1.15	0.0020	0.040
23	hsa-miR-376b	*MIR376B*	14q32.31		2.23	0.0021	0.040
24	hsa-miR-370	*MIR370*	14q32.31		1.77	0.0022	0.041
25	hsa-miR-134	*MIR134*	14q32.31		1.32	0.0023	0.041
26	hsa-miR-381	*MIR381*	14q32.31		1.63	0.0034	0.048
**B. RRMS patients in relapse versus RRMS patients in remission**							
1	hsa-miR-1	*MIR1-1*/ *MIR1-2*	20q13.33/18q11.2		3.82	3.58E-06	0.002

miRNA was considered as differentially expressed if *p*_adj_ < 0.05; −1< Log_2_FC >1.

**Table 3:** Post-hoс analysis of differential expression of miRNAs from *DLK1-DIO3* imprinted locus in peripheral blood mononuclear cells of relapsing-remitting multiple sclerosis (RRMS) patients compared to healthy controls (HCs) as determined by RT-*q*PCR (separately for men and women).

MiRNAs	Men: RRMS versus HCs (16 versus 10)		Women: RRMS versus HCs (20 versus 10)	
	Log_2_FC	*p*_adj_	Log_2_FC	*p*_adj_
miR-431	3.05	0.00014	0.16	0.99
miR-127-3p	3.00	0.00028	0.55	0.85
miR-379	3.06	0.0018	−0.90	0.48
miR-376c	2.50	0.00001	0.07	0.99
miR-381	2.52	0.00006	0.08	0.99
miR-410	2.49	0.00001	0.36	0.99
miR-656	2.81	0.0001	0.97	0.36

MiRNA expression was calculated relative to endogenous RNU6B using ΔΔCt method. Tukey-test was used to estimate statistical significance between compared groups. MiRNA considered to be differentially expressed if −1 ≤ Log_2_FC ≥1 and *p*_adj_ < 0.05 (values are given in bold).

## Supplementary Materials

The following are available online at http://www.mdpi.com/2073-4409/8/2/133/s1, Table S1: The high-throughput sequencing data on miRNA differential expression separately in men and women, Table S2: Checking the assumptions for two-way ANOVA. Table S3: Post-hoc analysis of miRNA expression between four compared groups using Tukey’s HSD, Table S4: The high-throughput sequencing data on expression of miRNAs from *DLK1-DIO3* locus, Equation S1: The testing of statistical hypothesis of the equality of the number of edges in observed and random networks.
