# Supplementary material for: MiRNAs from DLK1-DIO3 Imprinted Locus at 14q32 are Associated with Multiple Sclerosis: Gender-Specific Expression and Regulation of Receptor Tyrosine Kinases Signaling
Source: Cells. 2019 Feb 8;8(2):133. doi: 10.3390/cells8020133 (PMC6406543; doi:10.3390/cells8020133)
Supplement: Supplementary file 1 [file cells-08-00133-s001.zip › Baulina et al. - Equitation S1.doc]

Equitation S1. The testing of statistical hypothesis of the equality of the number of edges in observed and random networks.

The significance of the connectivity of a given set of nodes is obtained by comparing the observed number of edges with the number expected for randomly distributed nodes of a set of the same cardinality obtained from 10^5^ simulations (modified from ). The resulting z-score is defined:

Where is the number of edges (cardinality of an edges set) and σ is the standard deviation.

For our case:

Z_score_ = 10.94 complies the p-value = 3.7 x 10^-28^ by the Normal distribution; it was implemented through function pnorm() in R v.3.5.1.

Thus, the null hypothesis of the equality of the number of edges in observed and random networks could be rejected.

1.	Ghiassian S D, Menche J, Chasman D I, Giulianini F, Wang R, Ricchiuto P, Aikawa M, Iwata H, Muller C, Zeller T, Sharma A, Wild P, Lackner K, Singh S, Ridker P M, Blankenberg S, Barabasi A L, Loscalzo J (2016) Endophenotype Network Models: Common Core of Complex Diseas*e*s. Sci Rep 6: 27414.
